# Detection of grey matter microstructural substrates of neurodegeneration in multiple sclerosis

**DOI:** 10.1093/braincomms/fcad153

**Published:** 2023-05-24

**Authors:** Eva A Krijnen, Andrew W Russo, Elsa Salim Karam, Hansol Lee, Florence L Chiang, Menno M Schoonheim, Susie Y Huang, Eric C Klawiter

**Affiliations:** Department of Neurology, Massachusetts General Hospital, Harvard Medical School, Boston, MA 02114, USA; MS Center Amsterdam, Anatomy and Neurosciences, Amsterdam Neuroscience, Amsterdam UMC location VUmc, 1081 HV Amsterdam, The Netherlands; Department of Neurology, Massachusetts General Hospital, Harvard Medical School, Boston, MA 02114, USA; Department of Neurology, Massachusetts General Hospital, Harvard Medical School, Boston, MA 02114, USA; Department of Radiology, Athinoula A. Martinos Center for Biomedical Imaging, Massachusetts General Hospital, Harvard Medical School, Charlestown, MA 02129, USA; Department of Radiology, Athinoula A. Martinos Center for Biomedical Imaging, Massachusetts General Hospital, Harvard Medical School, Charlestown, MA 02129, USA; MS Center Amsterdam, Anatomy and Neurosciences, Amsterdam Neuroscience, Amsterdam UMC location VUmc, 1081 HV Amsterdam, The Netherlands; Department of Radiology, Athinoula A. Martinos Center for Biomedical Imaging, Massachusetts General Hospital, Harvard Medical School, Charlestown, MA 02129, USA; Department of Neurology, Massachusetts General Hospital, Harvard Medical School, Boston, MA 02114, USA

**Keywords:** multiple sclerosis, grey matter, neurodegeneration, advanced diffusion imaging

## Abstract

Multiple sclerosis features complex pathological changes in grey matter that begin early and eventually lead to diffuse atrophy. Novel approaches to image grey-matter microstructural alterations *in vivo* are highly sought after and would enable more sensitive monitoring of disease activity and progression. This cross-sectional study aimed to assess the sensitivity of high-gradient diffusion MRI for microstructural tissue damage in cortical and deep grey matter in people with multiple sclerosis and test the hypothesis that reduced cortical cell body density is associated with cortical and deep grey-matter volume loss. Forty-one people with multiple sclerosis (age 24–72, 14 females) and 37 age- and sex-matched healthy controls were scanned on a 3 T Connectom MRI scanner equipped with 300 mT/m gradients using a multi-shell diffusion MRI protocol. The soma and neurite density imaging model was fitted to high-gradient diffusion MRI data to obtain estimates of intra-neurite, intra-cellular and extra-cellular signal fractions and apparent soma radius. Cortical and deep grey-matter microstructural imaging metrics were compared between multiple sclerosis and healthy controls and correlated with grey-matter volume, clinical disability and cognitive outcomes. People with multiple sclerosis showed significant cortical and deep grey-matter volume loss compared with healthy controls. People with multiple sclerosis showed trends towards lower cortical intra-cellular signal fraction and significantly lower intra-cellular and higher extra-cellular signal fractions in deep grey matter, especially the thalamus and caudate, compared with healthy controls. Changes were most pronounced in progressive disease and correlated with the Expanded Disability Status Scale, but not the Symbol Digit Modalities Test. In multiple sclerosis, normalized thalamic volume was associated with thalamic microstructural imaging metrics. Whereas thalamic volume loss did not correlate with cortical volume loss, cortical microstructural imaging metrics were significantly associated with thalamic volume, and not with cortical volume. Compared with the short diffusion time (Δ = 19 ms) achievable on the Connectom scanner, at the longer diffusion time of Δ = 49 ms attainable on clinical scanners, multiple sclerosis-related changes in imaging metrics were generally less apparent with lower effect sizes in cortical and deep grey matter. Soma and neurite density imaging metrics obtained from high-gradient diffusion MRI data provide detailed grey-matter characterization beyond cortical and thalamic volumes and distinguish multiple sclerosis–related microstructural pathology from healthy controls. Cortical cell body density correlates with thalamic volume, appears sensitive to the microstructural substrate of neurodegeneration and reflects disability status in people with multiple sclerosis, becoming more pronounced as disability worsens.

## Teaser

Krijnen *et al*. evaluated a novel *in vivo* diffusion imaging model for grey-matter microstructural damage in people with multiple sclerosis using high-gradient MRI. Grey-matter cellular changes appear sensitive to detect the microstructural substrate of neurodegeneration and reflect clinical disability status.

## Graphical Abstract

**Graphical abstract:**
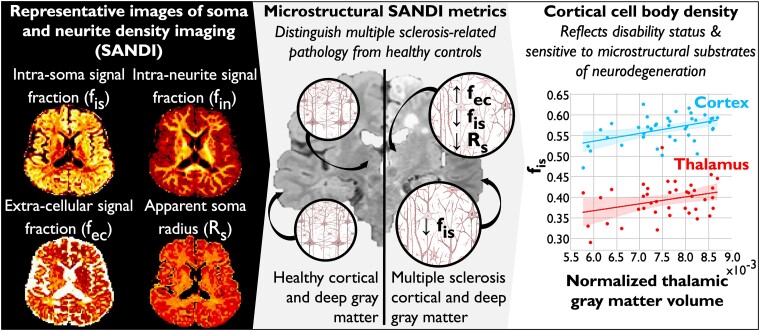

## Introduction

Multiple sclerosis (MS) is an inflammatory demyelinating and neurodegenerative disease of the CNS. In addition to characteristic white matter (WM) changes, MS features complex pathological changes in grey matter (GM), eventually leading to generalized atrophy. Whole-brain atrophy is a major determinant of clinical and cognitive worsening and is most pronounced in progressive MS (PMS).^1-3^ Whole-brain atrophy is used as a primary outcome measure in Phase 2 PMS clinical trials and is driven in part by neuroaxonal degeneration in the GM.^4-6^

While GM volume loss is an important predictor of clinical outcomes in people with MS and explains long-term disability better than WM atrophy,^7,8^ the exact microstructural underpinnings of atrophy and their temporal association with volume loss are still largely unexplored. Findings from histopathology and longitudinal imaging studies suggest that neurodegeneration begins at disease onset alongside neuroinflammation.^9,10^ Neuropathology studies have shown that chronic inflammation in the CNS plays a central role in the dysregulation of neuronal and axonal metabolism, resulting in neuroaxonal damage through apoptosis and necrosis, among other mechanisms.^11,12^ Neuroaxonal degeneration occurs not only at sites of overt demyelination but also throughout the brain parenchyma, including in normal-appearing (NA) tissue without evidence of focal inflammation.^13-15^

Neuronal and axonal loss is highly heterogeneous across GM but follows a reasonably consistent sequence throughout the disease course.^1,2,16^ In people with relapsing-remitting MS (RRMS), GM volume loss presents early in the posterior cingulate gyrus as well as in the precuneus and thalamus.^16^ In particular, volume loss in the thalamus and other deep GM nuclei, e.g. the caudate and putamen, have gained increased attention in recent years due to their relevance to clinical outcomes.^17-23^ Many studies have shown that the deep GM exhibits a disproportionate degree of MS-specific atrophy over time and is associated with an increased risk of disability progression.^3,24^ In people with MS, non-relapsing clinical progression may reflect a point beyond which compensatory mechanisms are no longer able to counteract the effects of irreversible neuroaxonal injury.^11,25^*In vivo* imaging markers of GM microstructural alterations prior to overt volume loss are needed to provide more sensitive monitoring of disease activity and progression prior to irreversible disability.

The advent of high-performance gradients on human MRI scanners has enabled the translation of promising diffusion-weighted (DW) microstructural imaging methods from animal and *ex vivo* studies to the investigation of tissue microstructure in the *in vivo* human brain.^26-32^ Current DW imaging modelling approaches are limited in their representation of GM microstructure as they cannot distinguish water in the extra-cellular space from water in cell bodies. To overcome this issue, a novel DW compartment-based model for apparent cell body, i.e. soma, and neurite density imaging (SANDI) has been proposed, in which the soma of any brain cell type is explicitly included as a spherical compartment of finite radius.^33^ The SANDI model was initially demonstrated in high-gradient diffusion MRI data from the MGH-USC Human Connectome Project and has been shown to define new contrasts reflecting brain cyto- and myelo-architecture.^33^ Another complication in the non-invasive characterization of tissue microstructure by DW-MRI is the concept of water exchange across the neurite membrane. It has been suggested that at reasonably short diffusion times (Δ < 20 ms), water exchange between neurites and soma, but also and more importantly, water exchange between neurites and the extra-cellular compartment can be considered negligible,^33-35^ supporting the importance of the high-gradient systems for characterizing microstructural changes in cortical and deep GM, although not yet available in clinical settings.

The goal of this study was to assess the sensitivity of SANDI on dedicated high-gradient diffusion MRI data acquired on the 3 T Connectome scanner for microstructural tissue alterations in cortical and deep GM in people with MS compared with healthy controls (HCs), and to evaluate microstructural tissue properties such as cell body and neurite density relative to GM volume, clinical disability scores and MS phenotype. For reference, we also assessed microstructural tissue alterations in NAWM and lesional WM in people with MS. We hypothesized that SANDI metrics, particularly cell body density in GM, would be reduced in cortical and deep GM of people with MS, reflecting cellular and neuronal loss in the MS brain, and that these metrics would be associated with GM volume loss. As SANDI and other DW microstructural models of GM gain traction in MS and other neurological diseases, we also sought to explore the impact of diffusion time on SANDI metrics in MS, taking advantage of the short diffusion times accessible on the Connectome scanner. We postulated that inter-compartmental water exchange would result in a shift of signal fractions from the intra-cellular to extra-cellular space at longer diffusion times, resulting in better sensitivity of SANDI at relatively short diffusion times (Δ = 19 ms) to cell body density compared with longer diffusion times (Δ = 49 ms). These short diffusion times are achievable with the use of cutting-edge hardware currently in development for the clinical setting.^36,37^

## Materials and methods

### Participants

This cross-sectional study was approved by the Massachusetts General Hospital institutional review board and is compliant with the Health Insurance Portability and Accountability Act guidelines. All participants provided written informed consent according to the Declaration of Helsinki. Forty-one people with a clinical diagnosis of MS who visited the MS Clinic at the Massachusetts General Hospital between 2015 and 2019 and 37 age- and sex-matched HC were included. Of the 41 people with MS, 32 had RRMS and 9 had PMS (3 primary- and 6 secondary-PMS). Inclusion criteria for people with MS were a diagnosis of clinically definite MS, being relapse free for at least 3 months, and receiving stable disease-modifying treatment or no treatment for at least 6 months. Exclusion criteria for all included participants were presence of other structural brain disease and contraindication to MRI. The current study includes a targeted re-analysis of data that were acquired on the Connectome scanner as part of a larger longitudinal imaging study in MS, and a subset of the data has been previously published with an emphasis on WM analyses.^30,38,39^

The following patient characteristics were extracted from the electronic medical records of the MS participants: age, sex, disease duration and type of disease-modifying therapy. All MS participants underwent a standard clinical examination within 1 week of the MRI scan. Neurological disability was assessed by a board-certified neurologist who was blinded to the imaging results using the Symbol Digit Modalities Test (SDMT) and Expanded Disability Status Scale (EDSS).

### Image acquisition

All participants underwent brain imaging in a 3 T MRI scanner (Magnetom Connectom; Siemens, Erlangen, Germany) equipped with a maximum gradient strength of 300 mT/m. A custom-built 64-channel-phased array head coil was used for signal reception.^40^ Diffusion data were acquired using a DW spin-echo single-shot EPI sequence in the sagittal plane [2 × 2 × 2 mm^3^ voxel size, echo time (TE)/repetition time (TR) = 77/3600 ms, simultaneous multi-slice imaging with slice acceleration factor 2, parallel imaging acceleration factor *R* = 2, anterior-to-posterior phase encoding, total acquisition time 51 min], following a previously established protocol.^30,41^ Non-DW images (*b* = 0) were acquired every 16 images. DW images were acquired at diffusion times of Δ = 19 ms using 8 *b*-values (*b* = 50–350–800–1500 s/mm^2^ in 32 directions, and *b* = 2400–3450–4750–6000 s/mm^2^ in 64 directions) and at Δ = 49 ms using 8 *b*-values (*b* = 200–950–2300 s/mm^2^ in 32 directions, and *b* = 4250–6750–9850–13 500–17 800 s/mm^2^ in 64 directions). For each diffusion time, a gradient pulse duration of 8 ms was used. Five *b* = 0 images with a reversed-phase encoding direction were separately acquired to correct for distortions due to susceptibility effects. Anatomical imaging included a T_1_-weighted multi-echo magnetization prepared rapid acquisition gradient echo (MEMPRAGE) sequence [1 × 1 × 1 mm^3^ voxel size, TE/TR/inversion time (TI) = 1.15–3.03–4.89–6.75 ms/2530 ms/1100 ms, *R* = 3, flip angle = 7°, acquisition time 3 min 58 s] and a 3D fluid-attenuated inversion recovery (FLAIR) sequence (0.9 × 0.9 × 0.9 mm^3^ voxel size, TE/TR/TI = 389 ms/5000 ms/1800 ms, *R* = 2, acquisition time 5 min 47 s).

### Diffusion MRI data processing

**Figure 1:**
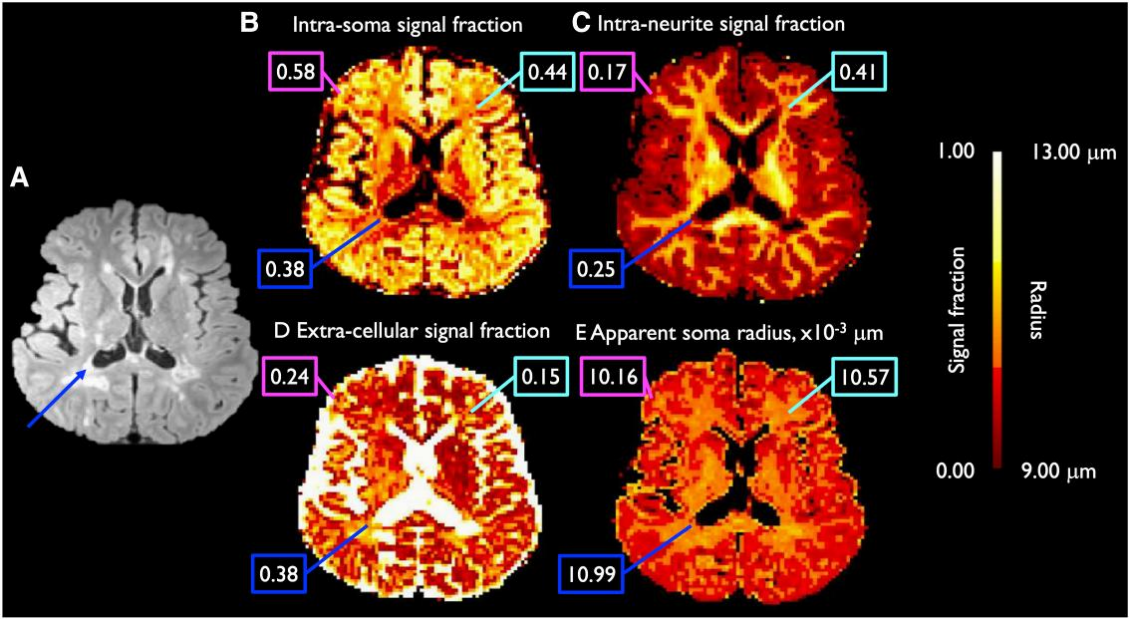
**Axial images of SANDI results.** Maps include the T_2_-weighted FLAIR image (**A**), showing WM lesions (dark blue arrow), of a representative patient with MS, and the corresponding intra-soma (**B**), intra-neurite (**C**) and extra-cellular (**D**) signal fractions and apparent soma radius (**E**; in μm). Values within a specific voxel consisting cortical GM (magenta; upper left boxes), NAWM (light blue; upper right boxes) and WM lesion (dark blue; lower left boxes) are displayed in boxes. Values displayed in images **B**, **C** and **D** are signal fractions ranging from 0.00 to 1.00, and are therefore unitless.

All DW images were pre-processed using an established pipeline.^41^ In brief, the magnitude DW images were acquired and corrected for gradient non-linearity using in-house software.^42^ Distortions in the DW images due to susceptibility and eddy current effects were corrected using topup (https://fsl.fmrib.ox.ac.uk/fsl/fslwiki/topup) and eddy (https://fsl.fmrib.ox.ac.uk/fsl/fslwiki/eddy) in the FMRIB Software Library (FSL version 5.0).^43-45^ We used the AMICO software^46^ (https://github.com/daducci/AMICO) to fit the SANDI model to the pre-processed diffusion data, applying a *λ*^2^ regularization term of 0.005 and the following default dictionary of atoms: intra-soma diffusivity = 3.0 × 10^−3^ mm^2^/s, soma radii = 1.56 × 10^−3^, 3.44 × 10^−3^, 4.44 × 10^−3^, 5.33 × 10^−3^, 6.00 × 10^−3^, 6.56 × 10^−3^, 8.11 × 10^−3^, 9.56 × 10^−3^, 1.17 × 10^−2^ mm, intra-neurite diffusivities = 9.17 × 10^−4^, 1.69 × 10^−3^, 3.00 × 10^−3^ mm^2^/s, and extra-cellular isotropic mean diffusivities = 3.61 × 10^−4^, 1.64 × 10^−3^, 3.00 × 10^−3^ mm^2^/s. The regularization factor was empirically chosen based on visual inspection of the fitting results for a range of regularization terms (0.0001 < *λ*^2^ < 0.05 in steps of 0.0005) and were in line with values used in previously published papers.^34^ We initially performed SANDI analysis on DW images acquired at diffusion time of Δ = 19 ms to avoid the potential confound of inter-compartmental exchange at diffusion times of Δ > 20 ms.^34,47^ Data acquired at diffusion time Δ = 49 ms were also fitted to SANDI and used to assess the impact of diffusion time on SANDI estimates. The model generated maps of the intra-neurite, extra-cellular and intra-soma signal fractions (*f*_in_, *f*_ec_ and *f*_is_), apparent soma radius (*R*_s_) and intra- and extra-neurite diffusivities (Fig. 1). Based on our hypotheses regarding cell body and neurite density in MS, we chose to include the SANDI metrics of *f*_in_, *f*_ec_, *f*_is_ and *R*_s_ for subsequent analyses. The diffusion tensor model was fitted on the pre-processed diffusion data of *b* = 800 s/mm^2^ using the *dtifit* function in FSL to derive maps of fractional anisotropy, which were used to improve the deep GM segmentation (see below).

### Registration, segmentation and imaging outcome determination

#### WM and GM segmentation

WM lesion segmentation of FLAIR hyperintense lesions was performed using a validated FreeSurfer-based automatic segmentation tool.^48^ A board- and subspecialty-certified neuroradiologist (12 years of experience) manually edited the lesion masks. Lesion volumes were calculated by multiplying lesion area by slice thickness.

Cortical surface and volumetric reconstruction were performed on the T1 MEMPRAGE data using FreeSurfer (version 5.3, http://surfer.nmr.mgh.harvard.edu). An experienced user manually reviewed these reconstructions and filled in WM lesions disrupting the cortical boundary to prevent misclassification of GM.^49^ After cortical surface reconstruction, we obtained a tissue segmentation map of cortical GM using the *5ttgen hsvs* function in MRtrix based on the surface reconstruction of FreeSurfer.^50,51^ GM and WM volume fractions were computed using the *mri_compute_volume_fractions* function of FreeSurfer. The cortical GM mask was multiplied by the GM volume fraction to derive a partial volume-weighted cortical GM mask, used in subsequent processing steps to minimize the potential bias from partial volumes.

Fourteen deep GM regions (seven in each hemisphere: accumbens, amygdala, caudate, hippocampus, pallidum, putamen and thalamus) were segmented using the Sequence Adaptive Multimodal SEGmentation^52^ (SAMSEG) of FreeSurfer, providing an enhanced T_1_-weighted image as input. The enhanced T_1_-weighted image was generated by in-house software combining the T_1_-weighted, FLAIR and fractional anisotropy images. This enhanced T_1_-weighted image has greater contrast between WM and GM, which can improve segmentation of deep GM regions. A separate FreeSurfer tool was used specifically for segmentation of the thalamus,^53^ proving the same enhanced T_1_-weighted image as input in order to better distinguish the lateral border of the thalamus from the surrounding WM. FLAIR images were missing in four HC subjects; hence, in these four subjects, SAMSEG was used providing just the T_1_-weighted and fractional anisotropy images as inputs. Segmentations were reviewed by an experienced rater for consistency.

To transform the partial volume-weighted cortical GM and WM maps, deep GM atlas and WM lesion mask to DWI space, DW images were first non-linearly registered to FreeSurfer space using ANTs registration,^54^ after which we applied the converted matrix and warp image to all maps. WM lesion segmentations in DW space were subtracted from the WM volume fraction map, yielding a partial volume-weighted NAWM map in DW space. Figure 1A shows the WMSA lesion segmentation in DWI space.

#### SANDI metric calculations

The cortical GM, NAWM and lesional WM masks and deep GM atlas in DWI space were overlaid on the maps for all SANDI microstructural metrics to calculate mean values of each metric per region of interest. Given the resolution of our DW images (2 mm isotropic), which was comparable with the average cortical thickness, we calculated partial volume-weighted mean values of cortical GM per microstructural metric to account for potential partial volume effects. This accentuated the contribution of voxels containing more cortical GM than voxels containing less GM.

#### Volume measures

Mean cortical GM volume and intra-cranial volume were calculated based on the T_1_-weighted images using the *mris_anatomical_stats* function of FreeSurfer. Deep GM volumes were calculated based on the deep GM segmentation in FreeSurfer space. All volumes are given in millilitre and corrected for intra-cranial volume, yielding normalized volume measures.

### Statistical analysis

Statistical analyses were performed with the use of Python v3.7 and IBM SPSS statistics 28.0 (SPSS Inc., Chicago, IL, USA). We used Kolmogorov–Smirnov testing and histogram inspection to test normality of the data. Participant characteristics were expressed as frequency with percentage, mean with standard deviation (SD), or median with inter-quartile range (IQR). Depending on the normality of the data, comparisons between groups were performed using independent samples *t*-tests or multivariate general linear models. The Mann–Whitney U-test was used as non-parametric alternative, if appropriate. All statistical tests were two-tailed. Effect sizes for SANDI metrics showing a significant difference between MS and HC groups were calculated based on Hedges’ *g*. *P*-values were false discovery rate (FDR)-corrected for multiple comparisons (*P*_corr_) per analysis step with *α* = 0.05.

#### Between-group changes in microstructure

Mean values of SANDI metrics were compared between people with MS and HC, separately for cortical and deep GM and NAWM. Deep GM SANDI metrics that showed near-significant differences, defined as *unadjusted P* < 0.10, were compared for each deep GM region (averaged between the two hemisphere) between people with MS and HC. In WM, near-significant SANDI metrics were compared between NAWM and lesional WM using paired *t*-tests.

#### Relation between microstructure and GM volume

In the regions showing significant differences in SANDI metrics between people with MS and HC, we further explored whether the metrics in specific regions of interest were related to volumes of those regions. First, normalized cortical and deep GM volumes were compared between people with MS and HC to determine the presence of atrophy in people with MS. Then, partial correlation coefficients, adjusted for age and sex, were calculated to assess the linear relation between mean SANDI metrics and normalized volumes in MS. Pearson’s or Spearman’s rank correlations were used depending on normality of the data.

#### Relation between microstructure and clinical disability

In addition, we sought to determine the relation between SANDI metrics in GM and WM, clinical disability and disease subtype. Therefore, SANDI metrics showing significant between-group differences (as described in the second paragraph) were further analysed among MS subtypes: HC versus RRMS, HC versus PMS and RRMS versus PMS. Also, significant correlations between SANDI metrics and GM volumes (as described in the third paragraph) were further assessed within RRMS and PMS separately.

In the total MS cohort, SANDI metrics showing significant between-group differences were correlated with EDSS and SDMT scores in people with MS. Linear correlations between SDMT scores and SANDI metrics were performed using Pearson’s partial correlations, adjusting for age and sex. Due to ordinal data, the rank correlation between EDSS and SANDI metrics was calculated based on Kendall’s Tau correlation. We also correlated SANDI metrics with age using Pearson’s partial correlation adjusting only for sex to assess the relationship of normal aging and potential measures of brain atrophy in MS.

#### Diffusion time differences

Data acquired at diffusion time Δ = 19 ms, which were used in all main analysis described above, were compared with data acquired at diffusion time Δ = 49 ms. To assess the diffusion time dependence of the SANDI metrics between groups and within the associations with GM volume, additional between-group analyses were performed using the data acquired at diffusion time Δ = 49 ms. Significant correlates of GM volume found at a diffusion time of Δ = 19 ms were assessed at Δ = 49 ms as well.

## Results

### Demographics

Table 1 shows the characteristics of included participants. Age and sex did not differ significantly between HC and people with MS. Our MS cohort consisted of predominantly people with RRMS [*N* = 33 (80.5%)] with a mean disease duration of <10 years. People with MS showed significant volume loss of both cortical and deep GM compared with HC [*t*(76) = 6.54, *P* = 6.39 × 10^−9^ and *t*(76) = 3.81, *P* = 2.82 × 10^−4^, respectively]. Of deep GM regions, all except the caudate and pallidum showed MS-related volume loss [*F*-range(1,76) = 5.51–24.12, *P*-range = 5.00 × 10^−6^–0.022], with the largest effect size seen in the thalamus (Hedges’ *g* = 1.10 compared with *g*-range = 0.11–0.62 in other deep GM structures). There were no missing data for any of the variables of interest.

**Table 1:** Baseline demographics and clinical characteristics of included participants

	Healthy volunteers (*N* = 37)	People with MS (*N* = 41)	Group differences^a^
			*P*-value*
Demographics			
Age, years	39.3 (14.3)	45.2 (12.9)	0.052
Sex, male/female [number (%)]	16 (43.2) / 21 (56.8)	11 (26.8) / 30 (73.2)	0.128
Education, years		16.2 (2.5)	
Disease characteristics			
Disease duration, years		9.7 (6.9)	
MS subtype, RR/SP/PP		33 (80.5)/5 (12.2)/3 (7.3)	
DMT use,^b^ yes [number (%)]		35 (85.4)	
Clinical characteristics			
EDSS, score		2.5 [2.0–3.5]	
SDMT, raw score		51.0 (12.5)	
MR characteristics			
WM lesion load, mL		4.13 [1.87–10.80]	
Cortical GM volume, mL	478.9 (57.8)	421.0 (73.9)	**2.68 × 10^−4^**
Normalized cortical GM volume, mL	0.30 (0.02)	0.27 (0.03)	**6.39 × 10^−9^**
Deep GM volume, mL	44.4 (4.0)	41.9 (5.0)	**0.016**
Normalized deep GM volume, mL	0.028 (0.002)	0.027 (0.002)	**2.82 × 10^−4^**

Variables are reported as mean (SD) or median (IQR) unless otherwise indicated. DMT, disease-modifying treatment; EDSS, Expanded Disability Status Scale; GM, grey matter; PP, primary progressive; MR, magnetic resonance; MS, multiple sclerosis; RR, relapsing-remitting; SDMT, Symbol Digit Modalities Test; SP, secondary progressive; WM, white matter. ^a^Independent samples *t*-test. ^b^Specific disease-modifying therapies include: nine dimethyl fumarate, eight glatiramer acetate, five fingolimod, five ocrelizumab, three interferon beta (1a/1b), two natalizumab, two rituximab and one teriflunomide. *P-values <0.05 are marked in bold.

The analysis reported in the next three sections focus on the short diffusion time (Δ = 19 ms) data, taking full advantage of the diffusion times accessible using the high-performance gradients on the Connectome scanner.^34,47^ The last section of the results, referred to as ‘Influence of diffusion time on SANDI metrics’, presents the SANDI analyses using the Δ = 19 ms and Δ = 49 ms data to examine systematically the diffusion time dependence of SANDI metrics in this population and provide results that may be more comparable with what is achievable on current clinical scanners.

### Between-group differences in tissue microstructure

Cortical *f*_is_ was reduced in people with MS compared with HC [*F*(1,76) = 5.10, *P* = 0.027, not surviving FDR correction; Table 2]. In deep GM, *f*_is_ was significantly decreased [*F*(1,76) = 6.53, *P*_corr_ = 0.039] and *f_ec_* significantly increased [*F*(1,76) = 6.43, *P*_corr_ = 0.039] in people with MS compared with HC. Region-specific analysis showed a significantly reduced *f*_is_ of the caudate and thalamus in people with MS compared with HC [*F*(1,76) = 13.91, *P*_corr_ = 0.007 and *F*(1,76) = 7.79, *P*_corr_ = 0.037, respectively]. In addition, *f*_ec_ of the caudate and thalamus were significantly increased, of which only differences in *f*_ec_ of the caudate survived FDR correction [*F*(1,76) = 12.36, *P*_corr_ = 0.007]. Among all deep GM nuclei, the pallidum was the only deep GM structure to show a significant difference in *R*_s_, which was significantly decreased in people with MS compared with HC [*F*(1,76) = 10.96, *P*_corr_ = 0.007; Supplementary Table 1]. Based on these results and previous literature suggesting a prominent role of the thalamus and caudate in disease progression and neurodegeneration in MS,^24,55^ we focused on these two regions in further analyses of SANDI metrics in the deep GM.

**Table 2:** Grey-matter SANDI metrics of included participants

	Δ = 19 ms					Δ = 49 ms				
	Healthy volunteers	People with MS	Group differences			Healthy volunteers	People with MS	Group differences		
	Mean (SD)	Mean (SD)	*F*-test^a^	*P*-value*	Effect size^b^	Mean (SD)	Mean (SD)	*F*-test^a^	*P*-value*	Effect size^b^
Cortical grey matter										
*f_in_*	0.17 (0.02)	0.17 (0.02)	*F*(1,76) = 1.50	0.224	0.28	0.11 (0.01)	0.11 (0.01)	*F*(1,76) = 1.54	0.22	0.28
*f_is_*	0.58 (0.03)	0.56 (0.03)	*F*(1,76) = 5.10	0.027	0.51	0.32 (0.01)	0.32 (0.02)	*F*(1,76) = 1.54	0.22	0.28
*f_ec_*	0.25 (0.02)	0.26 (0.04)	*F*(1,76) = 2.85	0.095	0.38	0.57 (0.01)	0.57 (0.02)	*F*(1,76) = 0.35	0.56	0.13
*R_s_*, μm	10.01 (0.08)	9.99 (0.12)	*F*(1,76) = 0.75	0.390	0.20	11.04 (0.12)	10.92 (0.27)	*F*(1,76) = 5.97	**0**.**017**	0.55
Deep grey matter										
*f_in_*	0.29 (0.02)	0.29 (0.02)	*F*(1,76) = 0.79	0.378	0.20	0.12 (0.01)	0.12 (0.01)	*F*(1,76) = 2.02	0.16	0.32
*f_is_*	0.52 (0.04)	0.50 (0.03)	*F*(1,76) = 6.53	**0**.**013**	0.58	0.33 (0.01)	0.33 (0.02)	*F*(1,76) = 4.43	0.039	0.48
*f_ec_*	0.20 (0.03)	0.22 (0.03)	*F*(1,76) = 6.43	**0**.**013**	0.58	0.55 (0.01)	0.55 (0.02)	*F*(1,76) = 2.35	0.13	0.35
*R_s_*, μm	10.20 (0.10)	10.14 (0.12)	*F*(1,76) = 4.60	0.035	0.49	10.11 (0.26)	9.91 (0.28)	*F*(1,76) = 10.40	**0**.**002**	0.73
Normal-appearing white matter										
*f_in_*	0.42 (0.03)	0.39 (0.04)	*F*(1,76) = 13.06	**5.40 × 10^−4^**	0.82	0.27 (0.02)	0.25 (0.03)	*F*(1,76) = 14.62	**2.68 × 10^−4^**	0.87
*f_is_*	0.43 (0.03)	0.44 (0.03)	*F*(1,76) = 3.63	0.061	0.43	0.26 (0.01)	0.27 (0.02)	*F*(1,76) = 7.11	**0**.**009**	0.60
*f_ec_*	0.15 (0.02)	0.16 (0.02)	*F*(1,76) = 9.34	**0**.**003**	0.69	0.46 (0.02)	0.48 (0.02)	*F*(1,76) = 10.13	**0**.**002**	0.72
*R_s_*, μm	10.23 (0.09)	10.22 (0.08)	*F*(1,76) = 0.24	0.627	0.11	10.33 (0.17)	10.36 (0.15)	*F*(1,76) = 0.41	0.53	0.14

GM SANDI values in healthy volunteers and people with MS are reported as mean (SD), separately for diffusion time Δ = 19 and 49 ms. GM, grey matter; NAWM, normal-appearing white matter; *f*_in_, intra-neurite signal fraction; *f*_is_, intra-soma signal fraction; *f*_ec_, extra-cellular signal fraction; *R_s_*, apparent soma radius. ^a^Multivariate general linear model. ^b^Effect sizes of significant changes between groups are based on Hedges’ *g*. *P-values <0.05 after FDR correction are marked in bold.

Regarding the WM, people with MS showed significantly decreased *f*_in_ and increased *f*_ec_ in NAWM compared with HC [*F*(1,76) = 13.06, *P*_corr_ = 0.004 and *F*(1,76) = 9.34, *P*_corr_ = 0.014, respectively]. In MS, *f*_is_ was slightly higher in NAWM compared with HC, but this increase did not reach statistical significance at the short diffusion time. Within people with MS, lesional WM showed lower *f*_in_, lower *f*_is_ and higher *f*_ec_ compared with NAWM [*t*(40) = 10.28, *P*_corr_ = 8.76 × 10^−13^, *t*(40) = 13.94, *P*_corr_ = 9.11 × 10^−17^, and *t*(40) = −15.52, *P*_corr_ = 4.74 × 10^−18^, respectively; Supplementary Table 2].

### Relationship between alterations in GM microstructure and volume

**Figure 2:**
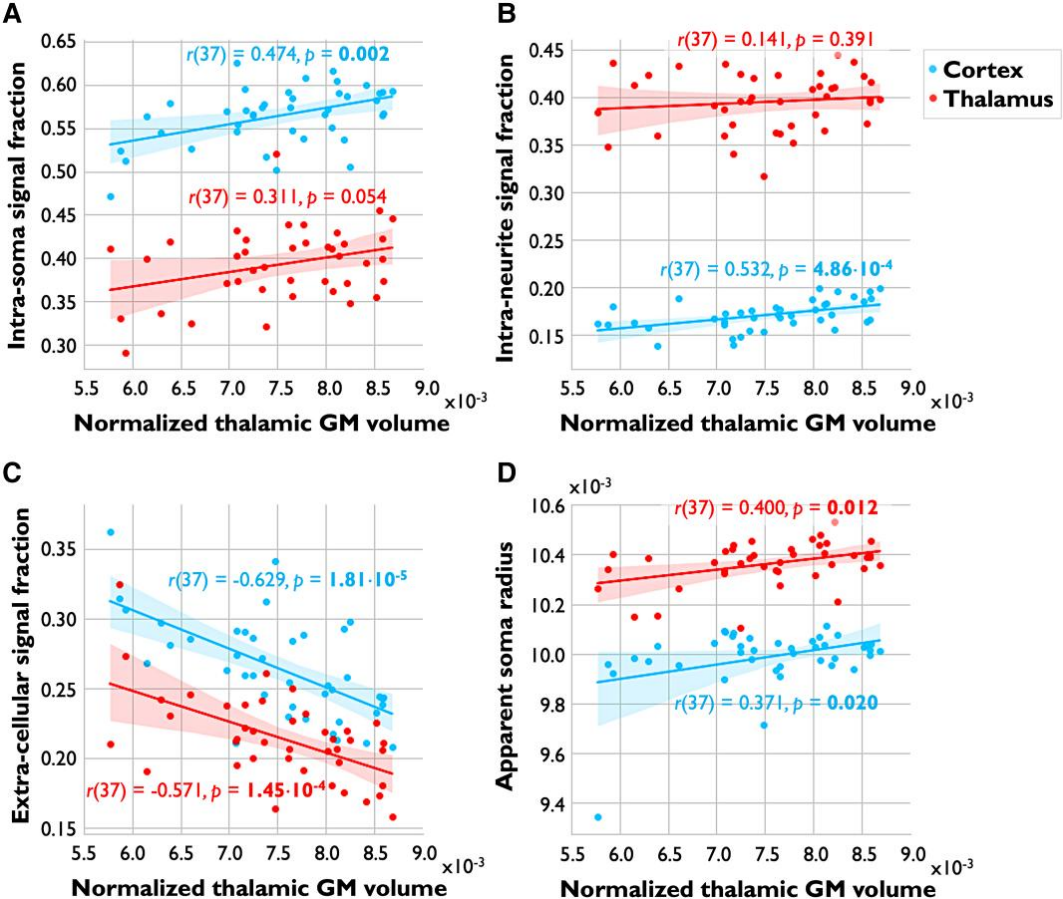
**Associations between SANDI metrics and thalamic volume.** Imaging metrics in the cortex (blue) and thalamus (red) are shown on the *y*-axes and normalized thalamic volume on the *x*-axes in people with MS. Intra-soma (**A**), intra-neurite (**B**) and extra-cellular signal fractions (**C**) and apparent soma radius (**D**; in μm) are shown in separate plots. Correlation coefficients with corresponding *P*-values are displayed in representative colours near each plot. *P-*values <0.05 surviving FDR correction are marked in bold. Values on the *y*-axis in subplot **A**, **B** and **C** are signal fractions ranging from 0.00 to 1.00, and are therefore unitless. As thalamic volumes were corrected for intra-cranial volume, yielding normalized volume measures, values on the *x*-axis are unitless as well. GM, grey matter.

To assess the relationship between MS-related alterations in cell body and neurite signal fractions and GM volume loss, the SANDI metrics that showed significant differences between MS and HC (see Table 2) were correlated with the corresponding normalized GM volume in people with MS. Cortical *f*_is_ and *f*_ec_ were not associated with normalized cortical volume in people with MS [*r*(37) = 0.061, *P* = 0.71, and *r*(37) = −0.062, *P* = 0.71, respectively]. In deep GM, people with MS showed a significant association between deep GM *f*_ec_ and deep GM volume [*r*(37) = 0.481, *P*_corr_ = 0.007]. Focusing on the thalamus, decreased thalamic *f*_is_ tended to be associated with decreased thalamic volume in people with MS [*r*(37) = 0.311, *P* = 0.054]. Normalized thalamic volume was significantly associated with increased thalamic *f*_ec_ [*r*(37) = −0.571, *P*_corr_ = 0.002] and decreased thalamic *R*_s_ [*r*(37) = 0.400, *P*_corr_ = 0.022; Fig. 2]. In the caudate, we found significant moderate associations between caudate volume and increased *f*_ec_ [*r*(37) = −0.407, *P*_corr_ = 0.022] and decreased *f*_is_ and *R*_s_ [*r*(37) = 0.405, *P*_corr_ = 0.022, and *r*(37) = 0.487, *P*_corr_ = 0.007, respectively; Supplementary Table 3].

The thalamus is known to be a major relay nucleus with extensive cortical anatomic connections. Axonal transection within WM lesions causes disconnections in these tracts projecting into and out of the cortex as well as the thalamus, likely contributing to the reduction in thalamic volume seen on histopathology.^19^ Thalamic volume loss may therefore reflect the net accumulation of MS-related neurodegenerative damage throughout the entire cortex. To assess the relationship between cortical and thalamic neurodegeneration in people with MS using the novel microstructural metrics presented here, normalized thalamic GM volumes were correlated with cortical SANDI metrics. MS-related cortical atrophy was not associated with normalized thalamic volume [*r*(37) = 0.264, *P* = 0.105]. However, thalamic volume was significantly associated with cortical *f*_is_, *f*_in_ and *R*_s_ [*r*-range(37) = 0.371–0.532, *P*_corr_-range = 0.002–0.050] and inversely associated with cortical *f_ec_* [*r*(37) = −0.629, *P*_corr_ = 1.80 × 10^−4^; Fig. 2].

### Relationship between GM microstructure and clinical disability

Trends in SANDI metrics in cortical and deep GM were analysed by disease subtype, i.e. in people with RRMS and PMS. Compared with HC, people with RRMS as well as people with PMS showed similar patterns in SANDI metrics in both GM and WM, with larger effect sizes in PMS (Supplementary Table 4). SANDI metrics in cortex, deep GM and WM as well as normalized GM volumes did not differ *between* MS subgroups. Both people with RRMS and PMS showed similar associations between normalized GM volumes and SANDI metrics as in the total MS cohort, but with stronger correlations seen in PMS (Supplementary Table 3).

In people with MS, SANDI metrics showing significant differences between MS and HC were correlated with clinical characteristics and disability measures. Older age was significantly associated with decreased *f*_is_ in cortical GM and NAWM [*r*(38) = −0.418, *P*_corr_ = 0.042, and *r*(38) = −0.446, *P*_corr_ = 0.042, respectively]. Increased EDSS as indicator of disease severity was associated with decreased cortical *f*_is_ and increased *f*_ec_ [*r*(39) = −0.319, *P*_corr_ = 0.042 and *r*(39) = 0.327, *P*_corr_ = 0.042, respectively]. SDMT did not show significant associations with SANDI metrics in either GM or WM (Supplementary Table 5).

### Influence of diffusion time on SANDI metrics

The main contributors to the total signal fraction in the cortex and deep GM shifted from predominantly *f*_is_ at Δ = 19 ms (∼0.53 at Δ = 19 ms and ∼0.33 at Δ = 49 ms) to *f*_ec_ at Δ = 49 ms (∼0.24 at Δ = 19 ms and ∼0.56 at Δ = 49 ms) and in NAWM from mainly *f*_in_ and *f*_is_ at Δ = 19 ms (∼0.41 at Δ = 19 ms and ∼0.26 at Δ = 49 ms) to *f*_ec_ at Δ = 49 ms (0.16 at Δ = 19 ms and 0.48 at Δ = 49 ms; Table 2). At Δ = 49 ms, between-group alterations in cortical and deep GM SANDI metrics were less apparent with lower effect sizes, except for *R*_s_. *R*_s_ showed significant reduction in people with MS in both the cortex and deep GM at Δ = 49 ms [*F*(1,76) = 5.97, *P*_corr_ = 0.041 and *F*(1,76) = 10.40, *P*_corr_ = 0.008, respectively] when compared with Δ = 19 ms.

In NAWM, signal fractions at Δ = 49 ms showed MS-related alterations to a similar extent as at Δ = 19 ms, i.e. *f*_in_ being increased in MS compared with HC [*F*(1,76) = 13.06, *P*_corr_ = 0.006 at Δ = 19 ms and *F*(1,76) = 14.62, *P*_corr_ = 0.003 at Δ = 49 ms] and *f*_ec_ being decreased [*F*(1,76) = 9.34, *P*_corr_ = 0.018 at Δ = 19 ms and *F*(1,76) = 10.13, *P*_corr_ = 0.008 at Δ = 49 ms; Table 2]. Regarding correlates of GM volume, thalamic atrophy was associated with cortical microstructure at Δ = 49 ms to the same extent as at Δ = 19 ms (Supplementary Table 6).

## Discussion

In this work, we evaluated the sensitivity of SANDI, a novel compartment model for GM, for microstructural tissue alterations in cortical and deep GM in people with MS compared with HC and evaluated these changes relative to GM atrophy and MS phenotype. We fitted the SANDI model to high-gradient diffusion MRI data acquired with gradient strengths up to 300 mT/m, which enabled measurements at a relatively short diffusion time to avoid the confounds of inter-compartmental water exchange, as advised in the original implementation of SANDI.^33^ Our cohort of people with MS showed trends towards decreased *f*_is_ in both the cortex and deep GM, specifically in the caudate and thalamus, compared with HC. Lower cortical *f*_is_ and *f*_in_ were significantly associated with thalamic volume loss in MS.

SANDI has been proposed as a promising biophysical model that captures microstructural features in GM,^33,47,56^ discriminating not only axonal and dendritic processes as captured by other diffusion models but also cell bodies.^33^ The application of SANDI to high-gradient diffusion MRI data in MS enabled us to probe alterations in the density of cell bodies and cellular processes corresponding to heterogeneous and complex pathological processes in GM and WM. At a short diffusion time, our cortical SANDI estimates in healthy cortex are in good agreement with previous SANDI work in healthy mouse and human brains.^27,33,34,56^ Jelescu *et al*.^34^ showed cortical *f*_is_ tends to decrease with increasing diffusion time, which is also reflected in our data, showing a lower cortical *f*_is_ at Δ = 49 ms. The same applies to the slight increase in cortical *R*_s_ estimates with longer diffusion time, consistent with the analyses on diffusion time dependence and exchange by Jelescu *et al*.^34^ Our healthy WM estimates at Δ = 49 ms tended to be lower and more comparable with previous high-gradient SANDI work in mouse cortex than our estimates at Δ = 19 ms.^27^ A recent study by Margoni *et al*.^56^ evaluated the SANDI model in people with MS using diffusion data acquired at a longer diffusion time (Δ = 46.9 ms) on a clinical MRI scanner with maximum gradient strength of 80 mT/m. Their reported estimates in WM and GM are in line with our estimates at Δ = 49 ms.^56^

Cortical atrophy accompanying neuronal and axonal degeneration and demyelination represents one of the most reliable predictors of clinical disability progression in MS.^16,57,58^ Our findings indicate that microstructural DW imaging using high-performance gradients captures alterations in GM composition at the mesoscopic scale. This may offer a novel predictive biomarker of disability progression beyond cerebral volume loss, which would be expected to postdate neuronal loss and may limit its sensitivity. It highlights the importance of developing and validating imaging markers that are temporally sensitive to the earliest effects of neurodegeneration and response to neuroprotective therapies. In our cohort, people with MS exhibited significant volume loss of GM structures with the exception of the caudate and pallidum, with the largest effect sizes observed in the cortex (*g* = 1.47) and thalamus (*g* = 1.10). People with MS also showed lower GM *f*_is_ compared with HC, whereas the GM *f*_in_ did not differ, indicating a potential limitation of current DW microstructural models that only account for neurites, e.g. NODDI. Microstructural alterations in the cortex were overall less evident than in deep GM structures compared with HC, i.e. lower effect sizes of changes in SANDI metrics in the cortex compared with deep GM and fewer changes surviving FDR correction. The cortex is known to possess a diversity of cell sizes, density and distribution and is characterized by spatial variations in MS-related atrophy across cortical areas.^16,59,60^ Histologically, demyelinated cortex shows a reduced cell density of up to 20% compared with NA cortex and seems more extensive in progressive disease with long disease duration.^61^ Hence, *f*_is_ would be expected to be more clearly decreased in people with PMS, as suggested by the results found by Margoni *et al*.^56^ They showed a significant decrease in both cortical *f*_is_ and *f*_in_ in PMS compared with HC and RRMS,^56^ suggesting that these cortical SANDI metrics are sensitive to cortical pathology in more advanced stages of the disease, highlighting the promise and potential of this method in detecting neuronal and axonal loss in progressive disease. The heterogeneity of cellular composition and pathologic involvement across the cortex may have diminished our sensitivity to MS-related alterations in the cortex within our RRMS-predominant cohort using SANDI; nevertheless, we were able to detect trends of increased cortical *f*_is_ in MS compared with HC and associations between cortical *f*_is_ and deep GM volume loss in MS that point to the promise of microstructural DW imaging in identifying GM microstructural alterations early in the disease course. Larger longitudinal studies using high-resolution and high *b*-value imaging are needed to better characterize the alterations in cortical microstructure throughout the disease course.

In deep GM, decreases in *f*_is_ were driven by reduced *f*_is_ in the thalamus and caudate—regions that are known for a disproportionate degree of MS-specific atrophy over time.^24^ Interestingly, our population of predominantly RRMS did not show significant volume loss of the caudate compared with HC, whereas microstructural changes in cell body density were clearly detectable. Based on histology, deep GM demyelination is especially prominent in the caudate and medial and anterior thalamic nuclei, and these regions show significantly reduced neuronal density in non-demyelinated, i.e. NA GM, and to a greater extent in demyelinated GM.^62,63^ These findings support the observed reductions in *f_is_*, particularly in the caudate and thalamus. Studying these microstructural changes in the light of neurodegeneration, thalamic volume loss only trended towards association with decreased thalamic *f*_is_ in people with MS, whereas it was significantly positively correlated with thalamic *R*_s_. Recent histopathological observations by Mahajan *et al*.^64^ in a predominantly PMS cohort found that the loss of neurons in the thalamus did not occur in proportion to loss of thalamic volume. Comparing neuronal density in the thalamic nuclei with the least and most volume, Mahajan *et al*.^64^ found an overall reduction in neuronal density of 17.6% in the nuclei with the least volume, suggesting neurons were lost in excess to volume loss. This could explain the absence of a clear association between thalamic neuronal density, i.e. *f*_is_, and thalamic volume in our study. Interestingly, Mahajan *et al*.^64^ also found that excessive neuronal loss seemed to be driven by a decrease in density of smaller neurons (200–400 μm2) instead of larger neurons (>400 μm2), suggesting a preferential loss of smaller inter-neurons relative to the larger relay neurons. Theoretically, this would indicate a relative increase in the radius of the cell bodies in a voxel within the thalamus with declining thalamic volume, yielding a negative correlation between *R*_s_ and thalamic volume. Conversely, Vercellino *et al*.^62^ studied the inflammatory and neurodegenerative changes in deep GM in a population consisting of ∼50% RRMS patients and found reductions in neuronal size in both the caudate and thalamus that were associated with longer disease duration in people with MS. Given the high degree of MS-specific atrophy over time in the caudate and thalamus,^24^ these findings would suggest a lower neuronal radius, *R*_s_, with declining deep GM volumes, in line with our observations of a positive correlation between thalamic and caudate volume and their respective *R*_s_. Previous high-gradient diffusion MRI measurements using the Connectome scanner fitted to a multi-compartment model of axonal diameter revealed a clinically relevant increase in apparent axon diameter in the corpus callosum of people with MS,^65^ suggesting that information on axonal and cellular size and density may provide added information beyond what can be gleaned from conventional diffusion tensor imaging. Nevertheless, for a detailed comparison of *R*_s_ and cellular size, further experiments comparing MRI and histology are needed.

The observed relationship between thalamic microstructure and volume loss suggests thalamic SANDI metrics, specifically *R*_s_, might be relevant surrogate measures of irreversible thalamic tissue loss. Interestingly, thalamic volume loss in people with MS was not only related to tissue microstructure in the thalamus itself but also in the cortex. Thus, cortical microstructural changes, as reflected here by decreased *f*_is_, might occur in proportion to thalamic volume loss, which has been shown to be a marker of neurodegeneration in the early stages of MS.^16^ Current results would therefore support the theory that thalamic volume loss reflects the net accumulation of MS-related neurodegenerative damage throughout the brain, making it a sensitive and appealing biomarker in people with MS. The cross-sectional study design limits us in drawing bold conclusions about causality and the sequential order of events, which warrants longitudinal studies. The absence of a correlation between cortical SANDI metrics and cortical volume could also be due to the contribution of other cortical pathology associated with heterogeneous cell populations, e.g. within demyelinated cortex, as described above, thereby confounding the expected correlation between cortical *f*_is_ and volume loss. Also, in contrast to deep GM, we studied the cortex as a whole, instead of focusing on specific parts of the cortex, e.g. the precuneus and cingulate gyrus, which are known to show early atrophy.^16^ Future work that evaluates the cortex on a regional basis may strengthen the association between cortical volume loss and SANDI metrics.

In this study, assessment of the WM demonstrated that people with MS showed significantly decreased *f*_in_ and increased *f*_ec_ in NAWM when compared with HC especially in progressive phenotypes; these findings are in line with those reported by other recent studies.^56^ Histopathological studies support these results and have shown diffuse axonal damage in the NAWM of people with MS.^7,66^ We did not observe significant MS-related changes in NAWM *f*_is_ estimates, as seen in GM, consistent with prior studies.^56^ The absence of a significant change in *f*_is_ in NAWM might be explained by the co-existence of an inflammatory process including microglial activation and neurodegenerative processes including loss of oligodendrocytes,^66,67^ counterbalancing each other’s effect on *f*_is_. In MS, lesional WM showed significantly lower *f*_in_ and *f*_is_ compared with NAWM, which is consistent with previous literature.^56,66^ After the acute phase of inflammation dissipates, inactive WM lesions are characterized by severe demyelination and axonal loss, and there is near-complete absence of macrophages and microglia.^66^ This corresponds with current findings of a lower cell body density, i.e. lower *f*_is_, and fewer axons and dendrites, i.e. lower *f*_in_. Further, histopathological evidence suggests that diffuse axonal damage and chronic active WM lesions are more profound in people with PMS than in acute or relapsing disease.^7,67,68^ In this study, the degree of microstructural loss (i.e. decrease in NAWM *f*_in_ and lesional *f*_in_ and *f*_is_) between PMS and RRMS was not significantly different, which may be due to the small sample size of our cohort. Nevertheless, current findings support the hypothesis that a similar neuropathological process occurs in RRMS and PMS as a continuum in the disease course.^56,69^

Finally, we assessed the relevance of SANDI metrics in relation to clinical disability. We did not find significant differences in SANDI metrics nor GM volumes between people with RRMS and PMS, likely due the small size of our PMS cohort. However, we found significant associations between EDSS as an indicator of disease severity and SANDI metrics in cortical GM, even in this relatively mildly affected MS cohort (median EDSS of 2.5). Our findings are in good agreement with the findings of Margoni *et al*.,^56^ whose MS cohort consisted of more severely disabled people with MS with a median EDSS of 6.5. Our results support the hypothesis that loss of microstructural integrity occurs early in the disease, tracks closely with disability status and becomes more pronounced as disability worsens.

Our study is limited by its cross-sectional study design and small sample size. Furthermore, in this study, we did not evaluate cortical lesions due to the limited set of contrasts and spatial resolution available at 3 T sensitive to cortical demyelination. A regional evaluation to cortical pathology in relationship to cortical SANDI metrics is an important follow-up study and may increase the effect size of alterations compared with healthy individuals. Another limitation raises from the potential bias in the SANDI results due to partial volume effects, in particular with the cerebrospinal fluid. Although we tried to limit this bias by generating partial volume-weighted microstructural maps, partial volume effects might not be fully eliminated, especially given the resolution of the DW images. Future work might harness free-water elimination methods to mitigate this bias. In our study, we analysed the magnitude DW images, which were available in all participants. Fitting the SANDI model to magnitude DW images may incur a bias in the estimation of intra-neurite signal fraction when compared with real-valued DW images due to the Rician noise distribution of magnitude data.^27^ Given that our main findings centred on the relationship between thalamic volume and cortical intra-cellular signal fraction, which is less affected by the Rician noise bias, we decided to make use of the data at our disposal and analyse the magnitude data in this study. Future work will focus on systematic examination of the differences in parameter estimation between magnitude and real-valued DW images in the subset of people of MS who had real-valued data available. The SANDI model has not yet been fully histopathologically validated in MS, having only been studied in a combined pathological MRI study evaluating the spinal cord of two people with MS and two non-neurological participants at 9.4 T.^70^ Both *f*_in_ and *f*_is_ showed significant correlations with the intensities of myelin and astrocyte staining, respectively. Further histopathological validation of the SANDI model in GM and WM in MS is needed to gain a better understanding of contributors to the derived metrics at different disease stages and within different tissues.

Assessment of neurodegeneration involving GM is clinically relevant and important for advancement of clinical trials. GM atrophy is widely used in clinical trials as an outcome measure for treatment response. However, the absolute rates for volume loss in the MS brain are small,^71^ requiring large sample sizes to demonstrate and complicated by the intra-subject variability in brain volume measures.^72^ This has hindered the uniform application of GM atrophy as an imaging biomarker in clinical and research settings. The increasing availability of high-performance gradients on clinical 3 T MRI scanners^41,73,74^ underscores the potential of advanced diffusion MRI models like SANDI for uncovering the microstructural substrate of neurodegeneration in MS and other neurological disorders, with the expectation that this would occur prior to GM volume loss. Further investigations of GM pathology using high-performance gradients are timely and necessary to assess the potential of DW microstructural metrics as highly sensitive measures of neuroprotection in clinical trials.

## Conclusion

Our study demonstrates that SANDI metrics obtained using high-gradient diffusion MRI are sensitive to cellular changes in the cortex and deep GM in the MS brain compared with healthy tissue, providing a detailed GM characterization that track with cortical and thalamic volumes. Application of *in vivo* biomarkers to detect early cellular loss could provide an advantage over assessment of volumetric changes in development of neuroprotective therapy in MS.

## Supplementary Material

fcad153_Supplementary_Data

## Data Availability

The data that support the findings of this study are available from the corresponding author, upon reasonable request.

## Abbreviations

DW =: diffusion-weighted
EDSS =: Expanded Disability Status Scale
*f*_ec_ =: extra-cellular signal fractions
*f*_in_ =: intra-neurite signal fractions
*f*_is_ =: intra-soma signal fractions
FDR =: false discovery rate
FLAIR =: fluid-attenuated inversion recovery
GM =: grey matter
HC =: healthy control
MEMPRAGE =: multi-echo magnetization prepared rapid acquisition gradient echo
MS =: multiple sclerosis
NA =: normal-appearing
PMS =: progressive multiple sclerosis
*R*_s_ =: apparent soma radius
RRMS =: relapsing-remitting multiple sclerosis
SAMSEG =: Sequence Adaptive Multimodal SEGmentation
SANDI =: soma and neurite density imaging
SDMT =: Symbol Digit Modalities Test
TE =: echo time
TI =: inversion time
TR =: repetition time
WM =: white matter
