# Supplementary material for: Detection of grey matter microstructural substrates of neurodegeneration in multiple sclerosis
Source: Brain Commun. 2023 May 24;5(3):fcad153. doi: 10.1093/braincomms/fcad153 (PMC10233898; doi:10.1093/braincomms/fcad153)
Supplement: fcad153_Supplementary_Data [file fcad153_supplementary_data.docx]

**Supplementary Table 1 Deep gray matter soma and neurite density imaging metrics in included participants**

|  | **Healthy volunteers** | **People with MS** | **Group differences** | | |
| --- | --- | --- | --- | --- | --- |
|  | **Mean (SD)** | **Mean (SD)** | ***F*-test^a^** | ***P*-value^b^** | **Effect size^c^** |
| **f_is_** | | | | | |
| Accumbens | 0.65 (0.04) | 0.64 (0.03) | *F*(1,76) = 1.43 | 0.235 | 0.27 |
| Amygdala | 0.55 (0.04) | 0.55 (0.04) | *F*(1,76) = 0.02 | 0.890 | 0.03 |
| Caudate | 0.57 (0.06) | 0.52 (0.06) | *F*(1,76) = 13.91 | **7.44⋅10^-4^** | 0.85 |
| Hippocampus | 0.49 (0.04) | 0.48 (0.03) | *F*(1,76) = 1.92 | 0.170 | 0.31 |
| Pallidum | 0.38 (0.05) | 0.35 (0.06) | *F*(1,76) = 3.99 | 0.049 | 0.45 |
| Putamen | 0.55 (0.04) | 0.53 (0.04) | *F*(1,76) = 4.07 | 0.047 | 0.46 |
| Thalamus | 0.42 (0.05) | 0.39 (0.04) | *F*(1,76) = 7.79 | **0.007** | 0.63 |
| **f_ec_** | | | | | |
| Accumbens | 0.15 (0.03) | 0.16 (0.02) | *F*(1,76) = 3.01 | 0.087 | 0.39 |
| Amygdala | 0.23 (0.03) | 0.23 (0.03) | *F*(1,76) = 0.02 | 0.876 | 0.04 |
| Caudate | 0.22 (0.06) | 0.27 (0.07) | *F*(1,76) =12.36 | **3.68⋅10^-4^** | 0.80 |
| Hippocampus | 0.28 (0.03) | 0.30 (0.03) | *F*(1,76) =3.43 | 0.068 | 0.42 |
| Pallidum | 0.16 (0.04) | 0.18 (0.05) | *F*(1,76) =3.54 | 0.064 | 0.43 |
| Putamen | 0.16 (0.03) | 0.17 (0.03) | *F*(1,76) =0.37 | 0.545 | 0.14 |
| Thalamus | 0.20 (0.04) | 0.21 (0.03) | *F*(1,76) =5.52 | 0.021 | 0.53 |
| **R_s_,** **μm** | | | | | |
| Accumbens | 10.17 (0.11) | 10.18 (0.08) | *F*(1,76) = 0.32 | 0.576 | 0.13 |
| Amygdala | 10.26 (0.07) | 10.30 (0.06) | *F*(1,76) = 5.15 | 0.026 | 0.51 |
| Caudate | 10.07 (0.14) | 10.01 (0.26) | *F*(1,76) = 1.25 | 0.267 | 0.25 |
| Hippocampus | 10.23 (0.13) | 10.22 (0.14) | *F*(1,76) = 0.13 | 0.720 | 0.08 |
| Pallidum | 10.08 (0.34) | 9.69 (0.63) | *F*(1,76) = 10.96 | **0.001** | 0.75 |
| Putamen | 10.22 (0.07) | 10.22 (0.06) | *F*(1,76) = 0.26 | 0.615 | 0.11 |
| Thalamus | 10.35 (0.10) | 10.36 (0.09) | *F*(1,76) = 0.51 | 0.480 | 0.16 |

MS = multiple sclerosis; f_is_ = intra-soma signal fraction; f_ec_ = extra-cellular signal fraction; R_s_ = apparent soma radius.

^a^Multivariate general linear model

^b^*P*-values < 0.05 after False Discovery Rate-correction are marked in bold.

^c^Effect sizes of significant changes between groups are based on Hedges’ g.

**Supplementary Table 2 White matter soma and neurite density imaging metrics in people with multiple sclerosis**

|  | **NAWM** | **Lesional WM** | **Group differences** | | |
| --- | --- | --- | --- | --- | --- |
|  | **Mean (SD)** | **Mean (SD)** | ***t*-test^a^** | ***P*-value^b^** | **Effect size^c^** |
| f_in_ | 0.39 (0.04) | 0.31 (0.06) | *t*(40) = 10.28 | **8.76⋅10^-13^** | 1.65 |
| f_is_ | 0.44 (0.03) | 0.38 (0.04) | *t*(40) = 13.94 | **6.07⋅10^-17^** | 2.00 |
| f_ec_ | 0.16 (0.02) | 0.33 (0.07) | *t*(40) = -15.52 | **1.58⋅10^-18^** | 3.17 |

NA = normal appearing; WM = white matter; f_in_ = intra-neurite signal fraction; f_is_ = intra-soma signal fraction; f_ec_ = extra-cellular signal fraction.

^a^Paired samples t-test

^b^*P*-values < 0.05 after False Discovery Rate-correction are marked in bold.

^c^Effect sizes of significant changes between groups are based on Cohen’s d.

**Supplementary Table 3 Associations between soma and neurite density imaging metrics and brain volumes**

|  | **People with MS** | | | **People with RRMS** | | | **People with PMS** | | |
| --- | --- | --- | --- | --- | --- | --- | --- | --- | --- |
|  | ***f_is_*** | ***f_ec_*** | ***R_s_*** | ***f_is_*** | ***f_ec_*** | ***R_s_*** | ***f_is_*** | ***f_ec_*** | ***R_s_*** |
|  | *r*(df)^a^ *p*-value^b^ | *r*(df)^a^ *p*-value^b^ | *r*(df)^a^ *p*-value^b^ | *r*(df)^a^ *p*-value^b^ | *r*(df)^a^ *p*-value^b^ | *r*(df)^a^ *p*-value^b^ | *r*(df)^a^ *p*-value^b^ | *r*(df)^a^ *p*-value^b^ | *r*(df)^a^ *p*-value^b^ |
| **Cortex** |  |  |  |  |  |  |  |  |  |
| Global volume | *r*(37)=0.061 *p*=0.712 | *r*(37)=-0.062 *p*=0.706 |  | - | - |  | - | - |  |
| **Deep GM** |  |  |  |  |  |  |  |  |  |
| Global volume | *r*(37)=0.266 *p*=0.101 | *r*(37)=-0.481 *p*=**0.002** | *r*(37)=0.263 *p*=0.105 | - | *r*(29)=-0.438 *p*=**0.014** | - | - | *r*(4)=-0.839 *p*=0.037 | - |
| Caudate | *r*(37)=0.405 *p*=**0.011** | *r*(37)=-0.407 *p*=**0.010** | *r*(37)=0.487 *p*=**0.002** | *r*(29)=0.373 *p*=0.039 | *r*(29)=-0.372 *p*=0.039 | *r*(29)=0.392 *p*=0.029 | *r*(4)=0.717 *p*=0.109 | *r*(4)=-0.681 *p*=0.136 | *r*(4)=0.912 *p*=**0.011** |
| Thalamus | *r*(37)=0.311 *p*=0.054 | *r*(37)=-0.571 *p*=**1.45⋅10^-4^** | *r*(37)=0.400 *p*=**0.012** | - | *r*(29)=-0.530 *p*=**0.002** | *r*(29)=0.512 *p*=**0.003** | - | *r*(4)=-0.883 *p*=**0.020** | *r*(4)=-0.323 *p*=0.533 |

Results of the associations between soma and neurite density imaging metrics and normalized volumes of cortex, global deep GM and caudate and thalamus in people with multiple sclerosis as well as multiple sclerosis subgroups are shown. Dash (-) denotes correlation was not calculated due to non-significance of main result. MS = multiple sclerosis; RR = relapsing-remitting; P = progressive; f_is_ = intra-soma signal fraction; f_ec_ = extra-cellular signal fraction; R_s_ = apparent soma radius; GM = gray matter.

^a^Partial correlation adjusted for age and gender, shown as the correlation coefficient (r) with degrees of freedom (df)

^b^Significant p-values surviving False Discovery Rate-correction are marked in bold

**Supplementary Table 4 Soma and neurite density imaging metrics in healthy volunteers and multiple sclerosis subgroups**

|  | **HC** | **RRMS** | **PMS** | **HC vs RRMS** | | | **HC vs PMS** | | | | **RRMS vs PMS** | | | |  |
| --- | --- | --- | --- | --- | --- | --- | --- | --- | --- | --- | --- | --- | --- | --- | --- |
|  | **Mean (SD)** | **Mean (SD)** | **Mean (SD)** | ***F*-test^a^** | ***P*-value^b^** | **Effect size^c^** | ***F*-test^a^** | ***P*- value^b^** | | **Effect size^c^** | | ***F*-test^a^** | ***P*-value^b^** | **Effect size^c^** | |
| **Cortex** | | | |  | | | | |  | | | | | |  |
| f_is_ – global | 0.58 (0.03) | 0.57 (0.03) | 0.55 (0.03) | *F*(1,68) = 3.14 | 0.081 | 0.42 | *F*(1,43) = 6.37 | 0.015 | | 0.98 | | *F*(1,39) = 1.37 | 0.25 | 0.46 | |
| f_ec_ – global | 0.25 (0.02) | 0.26 (0.04) | 0.28 (0.03) | *F*(1,68) = 1.34 | 0.251 | 0.28 | *F*(1,43) = 6.54 | 0.014 | | 1.00 | | *F*(1,39) = 1.20 | 0.28 | 0.43 | |
| **Deep GM** | | | |  | | | | |  | | | | | |  |
| f_is_ – global | 0.52 (0.04) | 0.50 (0.03) | 0.48 (0.03) | *F*(1,68) = 3.97 | 0.050 | 0.48 | *F*(1,43) = 6.21 | 0.017 | | 0.97 | | *F*(1,39) = 2.04 | 0.16 | 0.56 | |
| f_is_ – caudate | 0.57 (0.06) | 0.53 (0.06) | 0.48 (0.05) | *F*(1,68) = 8.10 | **0.006** | 0.68 | *F*(1,43) =16.72 | **1.86⋅10^-4^** | | 1.59 | | *F*(1,39) = 5.74 | 0.021 | 0.94 | |
| f_is_ – thalamus | 0.42 (0.05) | 0.40 (0.04) | 0.38 (0.05) | *F*(1,68) = 5.13 | 0.027 | 0.54 | *F*(1,43) = 6.26 | 0.016 | | 0.98 | | *F*(1,39) = 1.65 | 0.21 | 0.51 | |
| f_ec_ – global | 0.20 (0.03) | 0.22 (0.03) | 0.22 (0.02) | *F*(1,68) = 4.44 | 0.039 | 0.50 | *F*(1,43) = 4.76 | 0.035 | | 0.85 | | *F*(1,39) = 0.66 | 0.42 | 0.32 | |
| f_ec_ – caudate | 0.22 (0.06) | 0.26 (0.07) | 0.31 (0.06) | *F*(1,68) = 7.50 | **0.008** | 0.66 | *F*(1,43) =16.37 | **2.13⋅10^-4^** | | 1.58 | | *F*(1,39) = 3.89 | 0.056 | 0.78 | |
| f_ec_ – thalamus | 0.20 (0.04) | 0.21 (0.03) | 0.22 (0.03) | *F*(1,68) = 3.89 | 0.053 | 0.47 | *F*(1,43) = 3.43 | 0.071 | | 0.72 | | *F*(1,39) = 0.54 | 0.47 | 0.29 | |
| R_s_ – global | 10.20 (0.10) | 10.14 (0.13) | 10.13 (0.10) | *F*(1,68) = 3.64 | 0.061 | 0.46 | *F*(1,43) = 2.80 | 0.102 | | 0.65 | | *F*(1,39) = 0.07 | 0.79 | 0.10 | |
| **White matter** | | | |  | | | | |  | | | | | |  |
| f_in_ – NAWM | 0.42 (0.03) | 0.39 (0.04) | 0.38 (0.02) | *F*(1,68) = 8.83 | **0.004** | 0.71 | *F*(1,43) =16.32 | **2.17⋅10^-4^** | | 1.57 | | *F*(1,39) = 0.96 | 0.33 | 0.45 | |
| f_in_ – lesional WM | - | 0.32 (0.06) | 0.29 (0.04) |  | - | - |  | - | | - | | *F*(1,39) = 1.82 | 0.19 | 0.53 | |
| f_is_ – NAWM | 0.43 (0.03) | 0.44 (0.03) | 0.45 (0.03) | *F*(1,68) = 2.59 | 0.112 | 0.39 | *F*(1,43) = 2.84 | 0.099 | | 0.66 | | *F*(1,39) = 0.35 | 0.56 | 0.23 | |
| f_is_ – lesional WM | - | 0.38 (0.04) | 0.36 (0.03) |  | - | - |  | - | | - | | *F*(1,39) = 1.55 | 0.22 | 0.49 | |
| f_ec_ – NAWM | 0.15 (0.02) | 0.16 (0.03) | 0.17 (0.02) | *F*(1,68) = 6.52 | **0.013** | 0.61 | *F*(1,43) = 9.89 | **0.003** | | 1.23 | | *F*(1,39) = 0.67 | 0.42 | 0.32 | |
| f_ec_ – lesional WM | - | 0.32 (0.07) | 0.37 (0.04) |  | - | - |  | - | | - | | *F*(1,39) = 3.65 | 0.063 | 0.75 | |

HC = healthy volunteers; RRMS = people with relapsing-remitting multiple sclerosis; PMS = people with progressive multiple sclerosis; f_is_ = intra-soma signal fraction; f_ec_ = extra-cellular signal fraction; f_in_ = intra-neurite signal fraction; GM = gray matter; NA = normal appearing; WM = white matter.

^a^Multivariate general linear model

^b^*P*-values < 0.05 after False Discovery Rate-correction are marked in bold.

^c^Effect sizes of significant changes between groups are based on Hedges’ g.

**Supplementary Table 5 Associations between cortical soma and neurite density imaging metrics and clinical characteristics in people with multiple sclerosis**

|  |  | **Age** | **EDSS** | **SDMT** |
| --- | --- | --- | --- | --- |
| **Cortex** |  | *r*(df)^a^, *p*-value^b^ | *r*(df)^c^, *p*-value^b^ | *r*(df)^d^, *p*-value^b^ |
|  | f_is_ | *r*(38) = -0.418, *p* = **0.007** | *r*(39) = -0.319, *p* = **0.005** | *r*(37) = 0.066, *p* = 0.689 |
|  | f_ec_ | *r*(38) = 0.242,  *p* = 0.133 | *r*(39) = 0.327, *p* = **0.004** | *r*(37) = -0.068, *p* = 0.679 |
| **Deep GM** | f_is_ | *r*(38) = -0.318,  *p* = 0.045 | *r*(39) = -0.147, *p* = 0.201 | *r*(37) = 0.036, *p* = 0.828 |
|  | f_ec_ | *r*(38) = 0.078,  *p* = 0.632 | *r*(39) = 0.157, *p* = 0.171 | *r*(37) = -0.138, *p* = 0.402 |
|  | R_s_ | *r*(38) = -0.111, *p* = 0.494 | *r*(39) = -0.249, *p* = 0.030 | *r*(37) = 0.162, *p* = 0.323 |
| **NAWM** | f_is_ | *r*(38) = -0.446, *p* = **0.004** | *r*(39) = 0.107, *p* = 0.349 | *r*(37) = -0.204, *p* = 0.214 |
|  | f_in_ | *r*(38) = 0.224, *p* = 0.165 | *r*(39) = -0.238, *p* = 0.038 | *r*(37) = 0.299, *p* = 0.064 |
|  | f_ec_ | *r*(38) = 0.192, *p* = 0.235 | *r*(39) = 0.204, *p* = 0.075 | *r*(37) = -0.244, *p* = 0.134 |

EDSS = Expanded Disability Status Scale; SDMT = Symbol Digit Modalities Test; GM = gray matter; NAWM = normal-appearing white matter; f_is_ = intra-soma signal fraction; f_in_ = intra-neurite signal fraction; f_ec_ = extra-cellular signal fraction; R_s_ = apparent soma radius.

^a^Partial correlation adjusted for gender, shown as the correlation coefficient (*r*) with degrees of freedom (df)

^b^Significant *p*-values surviving False Discovery Rate-correction are marked in bold

^c^Kendall’s Tau correlations (due to ordinal data), shown as the correlation coefficient (*r*) with degrees of freedom (df)

^d^Partial correlation adjusted for age and gender, shown as the correlation coefficient (*r*) with degrees of freedom (df)

**Supplementary Table 6 Associations between cortical soma and neurite density imaging metrics and caudate and thalamic volumes**

|  |  | **Δ = 19 ms** | | **Δ = 49 ms** | |
| --- | --- | --- | --- | --- | --- |
|  |  | **Thalamus** | **Caudate** | **Thalamus** | **Caudate** |
|  |  | Volume | Volume | Volume | Volume |
|  |  | *r*(df)^a^, *p*-value^b^ | *r*(df)^a^, *p*-value^b^ | *r*(df)^a^, *p*-value^b^ | *r*(df)^a^, *p*-value^b^ |
| **Cortex** | Volume | *r*(37) = 0.264, *p* = 0.105 | *r*(37) = 0.153, *p* = 0.354 |  |  |
|  | f_is_ | *r*(37) = 0.474**,** *p* = **0.002** | *r*(37) = 0.259, *p* = 0.111 | *r*(37) = 0.411, *p* = **0.009** | *r*(37) = 0.241, *p* = 0.14 |
|  | f_in_ | *r*(37) = 0.532**,** *p* = **4.86⋅10^-4^** | *r*(37) = 0.275, *p* = 0.090 | *r*(37) = 0.631**,** *p* = **1.65⋅10^-5^** | *r*(37) = 0.268 *p* = 0.099 |
|  | f_ec_ | *r*(37) = -0.629**,** *p* = **1.81⋅10^-5^** | *r*(37) = -0.333, *p* = 0.038 | *r*(37) = -0.563**,** *p* = **1.93⋅10^-4^** | *r*(37) = -0.286 *p* = 0.078 |
|  | R_s_ | *r*(37) = 0.371,  ***p* = 0.020** | *r*(37) = 0.173, *p* = 0.291 | *r*(37) = 0.445**,** *p* = **0.004** | *r*(37) = 0.273 *p* = 0.092 |

Results of the associations between cortical soma and neurite density imaging metrics and normalized caudate and thalamic volumes in people with multiple sclerosis at two different diffusion times (Δ = 19 and 49 ms) are shown.

f_is_ = intra-soma signal fraction; f_in_ = intra-neurite signal fraction; f_ec_ = extra-cellular signal fraction; R_s_ = apparent soma radius.

^a^Partial correlation adjusted for age and gender, shown as the correlation coefficient (*r*) with degrees of freedom (df)

^b^Significant *p*-values surviving False Discovery Rate-correction are marked in bold
